# Beyond Tumor-Intrinsic STAT3: Integrating Metabolic and Immune Communication in the Tumor Microenvironment of Cervical, Ovarian, and Endometrial Cancers

**DOI:** 10.3390/cancers18182960

**Published:** 2026-09-14

**Authors:** Larissa Fonseca Marques, Fabiane Cristina Colunna, Jordy Alexander Lasso Larco, Francisco Cândido do Nascimento Pombo, Ana Paula Lepique

**Affiliations:** 1 Department of Immunology, Institute of Biomedical Sciences, University of São Paulo (ICB-USP), São Paulo 05508-000, Brazil; larimarques98@usp.br (L.F.M.); fabiane.colunna@usp.br (F.C.C.); jordylarco12@gmail.com (J.A.L.L.); francisco.pombo@usp.br (F.C.d.N.P.); 2 Comprehensive Center for Precision Oncology, University of São Paulo, São Paulo 01246-903, Brazil; 3 National Institute of Science and Technology for Research in Infectious and Chronic Diseases of the Mucosa and Skin (INCT Mucosa and Skin), Universidade Federal de Minas Gerais, Belo Horizonte 31270-901, Brazil

**Keywords:** STAT3, tumor microenvironment, ovarian cancer, cervical cancer, endometrial cancer, metabolism, immunotherapy, immune regulation

## Abstract

Cervical, ovarian, and endometrial cancers comprise biologically distinct malignancies with substantial heterogeneity in their molecular alterations, metabolic dependencies, immune landscapes, and therapeutic responses. Despite advances in surgery, chemotherapy, radiotherapy, targeted therapies, and immunotherapy, these cancers remain major causes of cancer-related morbidity and mortality worldwide. Signal Transducer and Activator of Transcription 3 (STAT3) is frequently activated in these tumor types, where its biological and clinical relevance varies according to disease context, histological subtype, and molecular characteristics. In addition to tumor-intrinsic functions regulating proliferation, survival, angiogenesis, and stemness, STAT3 participates in dynamic interactions between malignant cells and the tumor microenvironment (TME), integrating inflammatory, metabolic, and stromal signals. Persistent STAT3 activation has been associated with immunosuppressive changes involving macrophage polarization, myeloid-derived suppressor cell expansion, dendritic cell dysfunction, regulatory T-cell accumulation, and impaired cytotoxic lymphocyte activity, although the extent and mechanisms of these effects differ among tumor types and experimental contexts. Metabolic alterations, including enhanced glycolysis, lactate accumulation, and hypoxia, may further interact with STAT3-dependent signaling and influence communication between tumor and stromal or immune compartments. In this review, we examine STAT3 as a signaling node linking tumor metabolism with immune regulation in cervical, ovarian, and endometrial cancers, while emphasizing disease- and subtype-specific differences. We summarize current evidence on STAT3-mediated tumor–microenvironment communication and discuss emerging STAT3-targeted therapeutic strategies. Finally, we highlight challenges related to biological heterogeneity, the lack of validated predictive biomarkers, and patient stratification that currently limit the clinical translation of STAT3-directed approaches.

## Simple Summary

Cervical, ovarian, and endometrial cancers are biologically heterogeneous diseases in which tumor progression is shaped not only by malignant cells but also by the surrounding tumor microenvironment. This complex environment is composed of immune cells, stromal cells, blood vessels, and metabolic factors that continuously communicate with tumor cells, promoting immune suppression, therapy resistance, and disease progression. Increasing evidence identifies Signal Transducer and Activator of Transcription 3 (STAT3) as multicompartmental and reciprocal signaling hub that integrates these inflammatory, metabolic, and stromal signals across multiple cellular compartments. In this review, we examine how STAT3 contributes to tumor–microenvironment communication in these three malignancies, with particular emphasis on the interplay between tumor metabolism and immune regulation. We also discuss emerging therapeutic strategies targeting STAT3 and highlight the challenges associated with disease heterogeneity, biomarker development, and patient stratification that must be addressed for successful clinical translation.

## 1. Introduction

**Figure 1:**
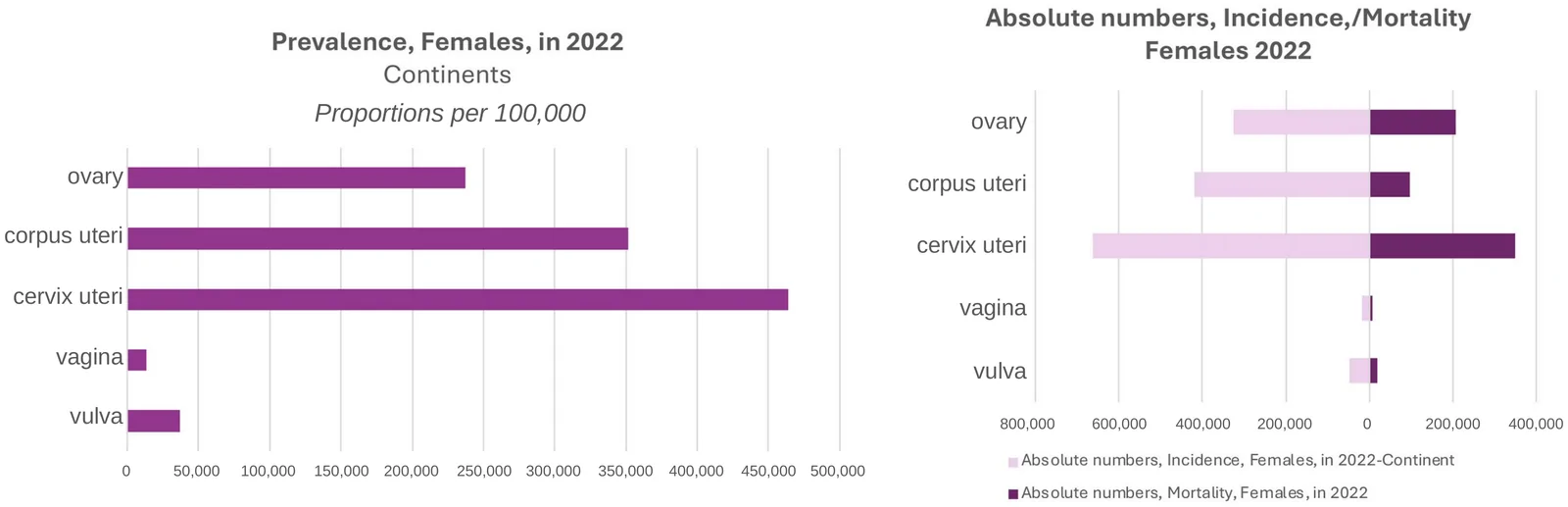
Global prevalence, incidence, and mortality of major gynecological cancers worldwide. Endometrial cancer is included under the category of corpus uteri. Data adapted from the International Agency for Research on Cancer (IARC), Global Cancer Observatory (GLOBOCAN 2022) [5].

Gynecological cancers comprise a heterogeneous group of malignancies originating from the female reproductive tract, including cervical, ovarian, endometrial, vulvar and vaginal cancers. Collectively, they represent one of the leading causes of cancer incidence and mortality among women worldwide (Figure 1) [1], although their epidemiology, molecular drivers and clinical behavior differ substantially. In this review, we focus specifically on cervical, ovarian and endometrial cancers, for which evidence regarding STAT3-mediated metabolic, immune and stromal communication is more extensively characterized. Cervical cancer remains one of the most frequently diagnosed malignancies in low- and middle-income countries, where limited access to HPV vaccination and screening programs continues to contribute to high mortality rates [2]. Conversely, endometrial cancer predominates in developed countries and is increasing incidence in parallel with obesity and metabolic disorders [3], whereas ovarian cancer accounts for a disproportionate number of gynecological cancer-related deaths owing to its asymptomatic progression, late diagnosis and frequent therapeutic resistance [4].

Despite these differences, cervical, ovarian and endometrial cancers share several biological features that contribute to disease progression, including chronic inflammation, metabolic adaptation, immune evasion and extensive communication between tumor cells and their surrounding microenvironment. Increasing evidence indicates that these processes do not develop independently but rather emerge from continuous interactions between malignant cells, stromal populations and infiltrating immune cells [6]. Understanding the molecular pathways coordinating these interactions has therefore become a central objective in the development of more effective therapeutic strategies.

Among these pathways, STAT3 (Signal Transducer and Activator of Transcription 3) has attracted considerable attention. Initially characterized as a transcription factor activated downstream of cytokines and growth factors, STAT3 is now recognized as an important regulator of numerous biological processes associated with cancer progression, including cell proliferation, apoptosis resistance, epithelial–mesenchymal transition, angiogenesis, invasion and stemness [7]. Constitutive STAT3 activation is reported in multiple solid tumors and hematological malignancies, where elevated STAT3 activity frequently correlates with poor clinical outcomes and resistance to conventional therapies [8]. The interest in STAT3 as a therapeutic target is therefore well justified. Over the past two decades, substantial efforts have been devoted to developing strategies capable of inhibiting STAT3 signaling through diverse approaches, including upstream kinase inhibition, disruption of STAT3 dimerization, antisense oligonucleotides, and, more recently, targeted protein degradation [8]. Parallel advances in molecular pathology have demonstrated persistent STAT3 activation in specific cervical, ovarian, and endometrial cancer contexts, although the extent and biological significance of STAT3 activation may vary according to histological and molecular subtype [9,10,11,12]. Nevertheless, accumulating evidence has also highlighted the remarkable complexity of STAT3 biology. Many of the biological processes influenced by STAT3—including inflammation, metabolic reprogramming, immune suppression and angiogenesis—are regulated by interconnected signaling networks involving NF-κB, PI3K/AKT, HIF-1α, MAPK and TGF-β, among others [13,14]. Rather than functioning independently, these pathways cooperate extensively, generating robust regulatory circuits that enable tumors to adapt to environmental stress while resisting therapeutic interventions. Within this network, STAT3 occupies a particularly interesting position because its activation extends beyond malignant epithelial cells to encompass multiple stromal and immune populations that constitute the tumor microenvironment. This broader perspective has shifted the way STAT3 is viewed in oncology. Instead of considering STAT3 exclusively as a tumor-cell signaling pathway, recent studies further support its participation in reciprocal communication between malignant cells, cancer-associated fibroblasts, endothelial cells, myeloid populations and lymphocytes [15]. The result is a dynamic signaling network in which persistent STAT3 activation may contribute to maintaining chronic inflammation, metabolic adaptation and immune dysfunction throughout the tumor microenvironment.

Metabolic reprogramming represents an important component of this network. Although altered glucose metabolism has traditionally been viewed as a hallmark of malignant cells, it is increasingly recognized that metabolites function as signaling molecules capable of influencing immune cell differentiation and function [16]. Lactate accumulation, hypoxia, nutrient competition and alterations in amino acid metabolism collectively shape the immune landscape of tumors through mechanisms involving multiple signaling pathways, including STAT3. Consequently, metabolism should no longer be regarded solely because of malignant transformation but also as an active participant in tumor–microenvironment communication [17].

These observations are particularly relevant for cervical, ovarian and endometrial cancers, where the composition of the local microenvironment differs substantially among tumor types. Ovarian cancer commonly develops within a peritoneal ecosystem that, in advanced-stage disease, is frequently marked by malignant ascites, abundant myeloid infiltrates and profound metabolic alterations, although ascites is not present in all patients and a substantial proportion present with solid peritoneal or extra-abdominal disease instead [18]. Importantly, ovarian cancer encompasses biologically distinct histological subtypes, and much of the mechanistic evidence discussed in this review is derived from specific subtypes or experimental models rather than ovarian cancer as a single entity. Cervical cancer evolves in the context of persistent HPV infection, chronic inflammation and progressive immune remodeling [2]. Endometrial cancer is likewise molecularly heterogeneous, and differences in molecular subtype may influence immune composition, metabolic dependencies and the relevance of STAT3 signaling. Molecular characterization has established four major subgroups: POLE-mutated (POLEmut), mismatch repair-deficient (MMRd), no specific molecular profile (NSMP), and p53-abnormal (p53abn), that differ substantially in genomic features, clinical behavior, and prognosis [19,20]. These molecular distinctions are also associated with substantial heterogeneity in the tumor immune microenvironment, with POLEmut and MMRd tumors generally exhibiting stronger immune infiltration, whereas immune-low phenotypes are more frequently observed among p53abn and NSMP tumors, although considerable variation exists within each molecular subgroup [21]. In parallel, endometrial cancer is strongly associated with metabolically dysregulated states, particularly obesity and insulin resistance, which may provide distinct metabolic contexts for tumor development and progression [22]. Accordingly, STAT3-associated inflammatory, immune, and metabolic programs should be interpreted within the molecular and biological context of individual endometrial cancer subgroups rather than extrapolated uniformly across the disease.

In this review, we discuss STAT3 within the broader context of the tumor microenvironment of cervical, ovarian and endometrial cancers, with emphasis on disease- and subtype-specific evidence when available. We first summarize canonical and non-canonical STAT3 signaling before examining its participation in metabolic adaptation and immune regulation across malignant and stromal compartments. We then discuss current advances in STAT3-targeted therapies and propose that considering STAT3 as an important signaling node linking tumor metabolism, immunity and stromal interactions provides a useful framework for developing rational combination strategies, while recognizing the biological heterogeneity that may influence the response to STAT3-directed interventions across these malignancies.

## 2. STAT3: From Signaling Pathway to Integrator of the Tumor Microenvironment

Signal Transducer and Activator of Transcription 3 (STAT3) belong to the STAT family of transcription factors, which comprises seven structurally related proteins (STAT1, STAT2, STAT4, STAT5A, STAT5B and STAT6) that regulate embryonic development, tissue homeostasis, immune responses and cell differentiation [23]. Among them, STAT3 has attracted particular attention in oncology because of its persistent activation in a broad spectrum of solid tumors and hematological malignancies [24]. While transient STAT3 activation is essential for normal physiological responses, constitutive signaling has been consistently associated with tumor progression, immune dysfunction and resistance to therapy [25].

**Figure 2:**
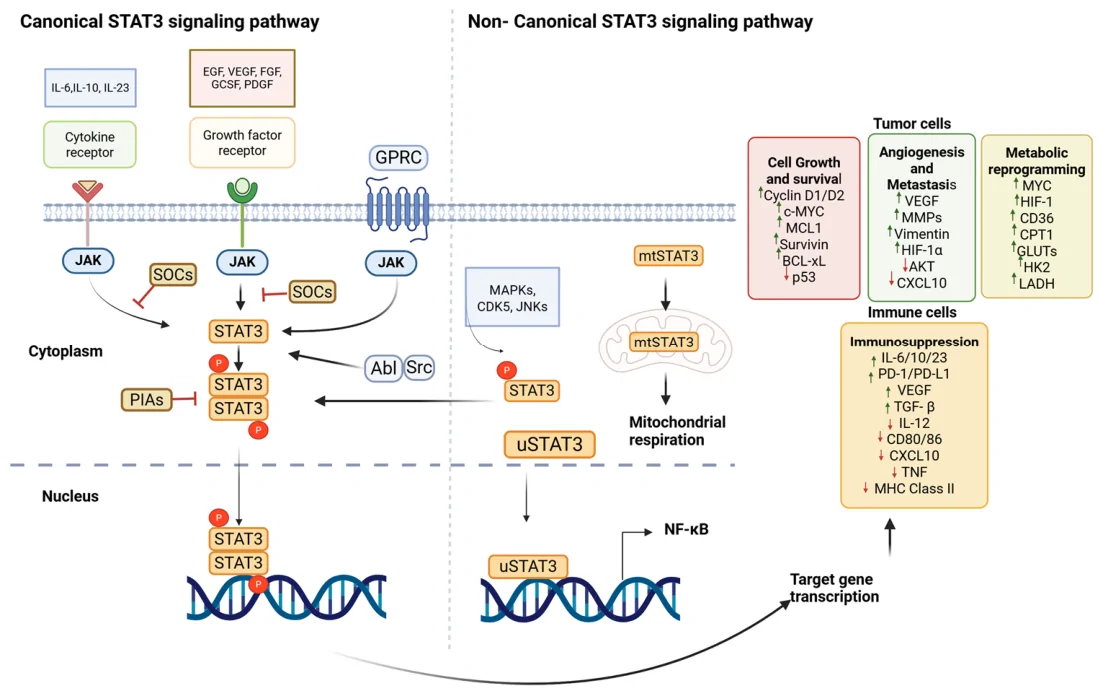
Schematic representation of the STAT3 signaling pathway and transcriptional regulation of target genes in tumor and immune cells. Cytokines such as interleukin-6 (IL-6) and growth factors such as epidermal growth factor (EGF) bind to their respective receptors, inducing conformational changes and activation of Janus kinase (JAK) family members. STAT3 is phosphorylated at Y705 by non-receptor tyrosine kinases (NRTKs), such as JAKs, and can also be activated by non-receptor tyrosine kinases such as Src and Abl. STAT3 dimerization promotes its translocation to the nucleus, where it functions as a transcription factor. STAT3 activation induces the transcription of target genes involved in tumor cell proliferation and survival, angiogenesis, metastasis, and immunosuppression (e.g., cyclin D1, survivin, Bcl-xL, VEGF; green arrows indicate upregulated targets and red arrows downregulated targets). Non-canonical STAT3 signaling includes Ser727 phosphorylation by serine/threonine kinases such as CDK5, JNKs, and MAPKs, mitochondrial STAT3 (mtSTAT3), and unphosphorylated STAT3 (uSTAT3). Non-canonical STAT3 signaling can influence cellular metabolism, mitochondrial respiration, NF-κB-dependent transcription, and genes associated with epithelial–mesenchymal transition (EMT). STAT3 signaling is negatively regulated by protein families such as protein inhibitors of activated STATs (PIAS) and suppressors of cytokine signaling (SOCS). PIAS proteins inhibit STAT3 binding to DNA, whereas SOCS proteins can inhibit JAK activity, promote JAK degradation, or interfere with STAT3 recruitment to cytokine receptors. Created in BioRender. Larco, J.A.L. (2026); https://BioRender.com/r0t2sc3.

Under physiological conditions, STAT3 activation is initiated by a variety of extracellular stimuli, including cytokines, growth factors and hormones. Members of the IL-6 family represent the best-characterized activators, although interferons, epidermal growth factor (EGF), vascular endothelial growth factor (VEGF), platelet-derived growth factor (PDGF), hepatocyte growth factor (HGF), non-receptor tyrosine kinases and GPCR-associated signaling can also result in STAT3 activation (Figure 2) [26]. Ligand binding induces receptor oligomerization and activation of Janus kinases (JAKs) or receptor-associated tyrosine kinases, promoting phosphorylation of STAT3 at tyrosine 705. Phosphorylated STAT3 forms homo- or heterodimers through reciprocal SH2-domain interactions and rapidly translocate to the nucleus, where it regulates transcription of genes involved in proliferation, survival, angiogenesis, inflammation and immune regulation [27] (Figure 2). STAT3 signaling is negatively regulated by SOCS proteins, which suppress JAK activity, and by PIAS proteins, which inhibit STAT3-dependent transcription [24].

Beyond this canonical mechanism, STAT3 also exerts important non-canonical functions. Alternative phosphorylation at serine 727, acetylation, methylation and other post-translational modifications influence transcriptional activity, mitochondrial function and interactions with additional signaling pathways [27]. Mitochondrial STAT3 (mtSTAT3) regulates oxidative phosphorylation, reactive oxygen species production and cellular metabolism, whereas unphosphorylated STAT3 (uSTAT3) participates in transcriptional regulation through interactions with transcription factors such as NF-κB (Figure 2) [28,29]. These non-canonical functions expand the biological role of STAT3 beyond conventional gene regulation and connect intracellular bioenergetics with adaptive transcriptional activity. In endometrial cancer, S727 phosphorylation has been reported in the absence of detectable Y705 phosphorylation, supporting a Y705-independent mode of STAT3 activity. In endometrial cancer cell models, inhibition of S727-phosphorylated STAT3 with HO-3867 reduced tumor cell viability and suppressed tumor growth, supporting a functional role for S727-dependent STAT3 signaling in this malignance [30]. On the other hand, in cervical cancer, non-canonical STAT3 activity appears more closely intertwined with canonical signaling, with S727 phosphorylation frequently occurring alongside Y705 phosphorylation and showing associations with HPV-related signaling and disease progression [31]. In platinum-resistant ovarian cancer cells, Jagged1/Notch signaling has been associated with increased STAT3 S727 phosphorylation, alongside enhanced epithelial–mesenchymal transition and invasive properties. Modulation of Jagged1 altered S727 phosphorylation and these phenotypes, suggesting that S727-dependent STAT3 signaling may contribute to platinum resistance in this context [32].

STAT3 activation has also been associated with EMT-related changes, including E-cadherin downregulation and increased expression of mesenchymal markers such as vimentin. These effects have been reported in experimental models of ovarian, cervical and endometrial cancer, although the underlying mechanisms and experimental contexts differ among malignancies. Collectively, canonical and non-canonical STAT3 signaling can contribute to multiple cancer-associated phenotypes. Across the cervical, ovarian and endometrial cancer models discussed below, STAT3 activation has been associated with tumor-cell proliferation, survival, invasion and metabolic adaptation, although the specific phenotypes and experimental contexts vary among malignancies and models. In immune cells, STAT3 signaling has likewise been implicated in macrophage polarization, dendritic-cell dysfunction, myeloid-derived suppressor-cell expansion and impaired antitumor immunity [25] (Figure 2). This functional versatility reflects the ability of STAT3 to integrate signals derived from cytokines, growth factors, metabolic stress and other signaling networks, positioning STAT3 as a central signaling hub within the tumor microenvironment.

In ovarian cancer models, STAT3 activation is positively associated with MMP-9 (Matrix Metalloproteinase-9) and MMP-2 (Matrix Metalloproteinase-2) expression [33,34], while STAT3 inhibition not only reduces cell migration but also decreases the invasive potential of endometrial cancer cells [35]. Constitutive STAT3 activation also induces epithelial–mesenchymal transition (EMT) by downregulating E-cadherin and upregulating mesenchymal markers such as vimentin, which are observed in ovarian, cervical and endometrial cancer [36,37,38]. In addition, STAT3 directly regulates transcriptional activator of vascular endothelial growth factor (VEGF) and hypoxia-inducible factor 1-alpha (HIF-1α) expression, which plays a central role in angiogenesis [39]. Inhibition of STAT3 activity reduces the expression of survivin, VEGF and vimentin, suppresses colony formation, viability, motility, and migration of resistant ovarian cancer cells [40]. Moreover, in vivo assays have shown that IL-6 leads to an increase in angiogenic activity in human cervical cancer cells, through VEGF upregulation [41].

The extensive crosstalk between STAT3 and other signaling networks is particularly relevant in cancer. Activation of PI3K/AKT, MAPK, NF-κB, TGF-β and HIF-1α pathways frequently occurs alongside STAT3 signaling, generating positive feedback loops that reinforce inflammation, metabolic adaptation and therapeutic resistance [13,14]. IL-6-mediated STAT3 activation, for example, cooperates with NF-κB to maintain chronic inflammatory programs, whereas hypoxia promotes reciprocal activation of HIF-1α and STAT3, enhancing angiogenesis and metabolic remodeling [13]. Likewise, interactions between STAT3 and PI3K/AKT signaling contribute to tumor cell survival, while communication with TGF-β signaling influences epithelial-to-mesenchymal transition, extracellular matrix remodeling and immune suppression [17]. These interactions highlight an important concept for interpreting STAT3 biology in cancer. The biological effects commonly attributed to STAT3—including proliferation, angiogenesis, invasion and immune escape—are not exclusive to this pathway; instead, they emerge from coordinated activity among multiple interconnected signaling networks.

### 2.1. STAT3 in Metabolic Reprogramming

Metabolic reprogramming is a hallmark of cancer that extends far beyond supporting the energetic and biosynthetic demands of rapidly proliferating tumor cells. Alterations in glucose, glutamine and lipid metabolism continuously reshape the tumor microenvironment (TME), generating conditions characterized by hypoxia, nutrient competition, extracellular acidification and the accumulation of bioactive metabolites. These metabolic changes influence not only malignant cells but also stromal and immune populations, thereby regulating macrophage polarization, dendritic cell function, lymphocyte activity and fibroblast behavior [42,43,44].

STAT3 occupies an important position within this metabolic–immune network by integrating environmental cues with transcriptional and mitochondrial responses that may support tumor adaptation. Beyond its canonical transcriptional activity, STAT3 regulates cellular metabolism through both nuclear and non-canonical mitochondrial functions. Mitochondrial STAT3 (mtSTAT3) contributes to oxidative phosphorylation (OXPHOS), reactive oxygen species (ROS) homeostasis and mitochondrial respiration, whereas nuclear STAT3 promotes transcriptional programs involved in glycolysis, hypoxic adaptation and metabolic plasticity [45].

Glutamine metabolism is extensively explored in cancer therapy, and recent evidence highlights a positive regulatory loop between STAT3 and the glutaminolysis pathway in various tumors [17]. A recent study shows that glutamine levels relate to ovarian cancer invasiveness by specifically modulating the STAT3 pathway [46]. In highly invasive OVCA cells, glutamine availability is required to maintain STAT3 phosphorylation at both Y705 and S727 residues. Moreover, expression of constitutively active STAT3 partially rescued growth inhibition induced by glutamine deprivation, supporting a functional contribution of STAT3 signaling to the cellular response to glutamine availability. Furthermore, mitochondrial STAT3 may contribute to redox homeostasis in malignant cells through non-transcriptional mechanisms [47]. Beyond its genomic activity, STAT3 manages the elevated production of mitochondrion-derived reactive oxygen species (ROS) typical of tumor transformation. In invasive cancer models, glutamine-derived glutathione (GSH) helps buffer oxidative stress, whereas inhibition of GSH synthesis reveal a vulnerability associated with mitochondrial STAT3 activity [47].

Curiously, investigations in cervical (SiHa and HeLa) and breast cancer (MDA-MB-231) models demonstrate that STAT3 has also been implicated in cellular responses to extracellular glutamine., coordinating cell proliferation independently of canonical Gln metabolism [48]. Glutamine deprivation induces metabolic stress and growth arrest, whereas STAT3 activation is associated with cellular recovery through regulation of targets including HIF-1α and c-Myc. These findings suggest that growth factor-mediated STAT3 activation may contribute to cellular adaptation to altered glutamine availability and potentially influence responses to strategies targeting glutamine uptake, suggesting that dual inhibition of STAT3 and Gln transporters could provide a more robust therapeutic outcome for these malignancies [48].

Moreover, STAT3 can transactivate genes that promote fatty acid oxidation, which may contribute to oxidative stress management and oxidative phosphorylation [49]. These observations may appear to contrast with evidence linking STAT3 to aerobic glycolysis; however, these metabolic effects are likely context-dependent and may coexist according to cellular state and environmental conditions. Notably, there is still limited evidence regarding the mitochondrial role of STAT3 in gynecological cancers. This gap may reflect the relatively small number of studies investigating compartment-specific STAT3 functions rather than the evidence against a role for mitochondrial STAT3 in these malignancies.

#### 2.1.1. Hypoxia and Metabolic Stress Reinforce Inflammatory Signaling

Progressive tumor growth inevitably generates regions of limited oxygen availability owing to inadequate vascularization and abnormal tissue architecture. Cellular adaptation to hypoxia is primarily coordinated by hypoxia-inducible factors (HIFs), which regulate angiogenesis, glucose metabolism, invasion and cell survival [50]. However, increasing evidence indicates that hypoxic adaptation depends on extensive interactions between HIF signaling and inflammatory pathways, including persistent STAT3 activation [50]. Reciprocal regulation between HIF-1 alpha and STAT3 has been described in several tumor models. STAT3 contributes to HIF-1 alpha transcriptional activity under hypoxic conditions, whereas HIF-dependent cytokine production further amplifies inflammatory signaling, generating positive feedback loops that sustain angiogenesis and metabolic adaptation [17]. This cooperation illustrates how metabolic stress is translated into coordinated transcriptional programs extending well beyond oxygen sensing alone. In ovarian cancer, hypoxia is particularly relevant because rapidly expanding peritoneal implants and malignant ascites generate spatially heterogeneous oxygen gradients that influence both tumor cells and infiltrating leukocytes [51]. Similarly, progressive vascular abnormalities during cervical cancer development contribute to localized hypoxia, further reinforcing inflammatory signaling [52]. Moreover, in endometrial cancer context, HIF-1 alpha expression is significantly more frequent in tumor than in normal endometrial tissue and correlates with higher histological grade, lymph node metastasis, myometrial invasion and reduced survival, indicating that hypoxic signaling is also clinically relevant in this tumor type [53]. These observations suggest that hypoxia should be considered as not only a consequence of tumor growth but also an active regulator of cellular communication within the tumor microenvironment.

#### 2.1.2. Lactate: From Metabolic By-Product to Immunoregulatory Mediator

Among the metabolites accumulating within tumors, lactate has emerged as one of the most influential regulators of the tumor microenvironment. Traditionally regarded as the product of aerobic glycolysis, lactate is now recognized as an active signaling molecule capable of influencing angiogenesis, extracellular matrix remodeling, immune cell differentiation and therapeutic responses [54].

High glycolytic activity combined with limited perfusion results in substantial lactate accumulation within many solid tumors, including ovarian and cervical cancers. Lactate is transported through monocarboxylate transporters (MCT1 and MCT4), allowing continuous metabolic exchange between neighboring cells while contributing to extracellular acidification [55]. These environmental changes influence virtually every component of the tumor microenvironment, extending the biological consequences of altered metabolism far beyond tumor cells themselves.

Growing evidence demonstrates that lactate affects immune populations through multiple complementary mechanisms. Elevated extracellular lactate impairs dendritic cells differentiation, reduces antigen presentation, limits cytotoxic T-cell activity and favors the development of regulatory immune populations [56]. Macrophages exposed to lactate acquire phenotypic characteristics associated with tissue remodeling and immune suppression, including increased production of IL-10, VEGF and other mediators that further reinforce chronic inflammation [57]. Although these responses involve several signaling pathways, including HIF-1 alpha, GPR81-mediated signaling and NF-κB, persistent STAT3 activation appears to participate in coordinating many of the downstream transcriptional programs associated with immunosuppressive macrophage polarization [54].

These observations are particularly relevant across cervical ovarian and endometrial cancers. Ovarian cancer ascites contains high concentrations of lactate together with abundant immunosuppressive myeloid cells, creating favorable conditions for persistent inflammatory signaling and impaired antitumor immunity [58]. In cervical cancer, HPV-driven metabolic reprogramming further contributes to lactate production while chronic inflammation sustains cytokine networks capable of reinforcing STAT3 activity [59]. Furthermore, in endometrial cancer, tumor cells display increased aerobic glycolysis and lactate release, which drives M2 polarization of tumor-associated macrophages; this M2 polarization correlates with deep myometrial invasion and advanced tumor stage. These lactate-stimulated M2 macrophages secrete elevated levels of IL-6, promoting epithelial–mesenchymal transition, angiogenesis, and invasiveness of endometrial cancer cells. Blockade of IL-6 signaling, as well as inhibition of the rate-limiting glycolytic enzyme LDHA, reduces these effects both in vitro and in a xenograft mouse model, and their combination with cisplatin further enhances the antitumor response [60]. Together, these findings suggest that lactate should not be viewed simply as a metabolic consequence of tumor growth but rather as an important component of the signaling network linking metabolism with immune regulation. Rather than activating STAT3 in isolation, lactate participates in a broader signaling network in which metabolic, inflammatory and stromal pathways cooperate to shape cellular behavior.

## 3. STAT3, the Tumor Microenvironment and Immune Responses in Cervical, Ovarian, and Endometrial Cancers

### 3.1. Stromal Cells Amplify STAT3-Dependent Communication Within the Tumor Microenvironment

The tumor microenvironment is maintained not only by malignant cells and infiltrating leukocytes but also by an extensive stromal compartment that actively contributes to tumor progression. Cancer-associated fibroblasts (CAFs), endothelial cells, mesothelial cells and adipocytes continuously exchange cytokines, growth factors, extracellular matrix components and metabolites with neighboring cells, creating a dynamic signaling network that supports chronic inflammation, tissue remodeling and immune dysfunction [15]. Rather than acting as passive structural elements, stromal cells actively influence tumor evolution and frequently represent important sources of persistent STAT3 activation.

**Figure 3:**
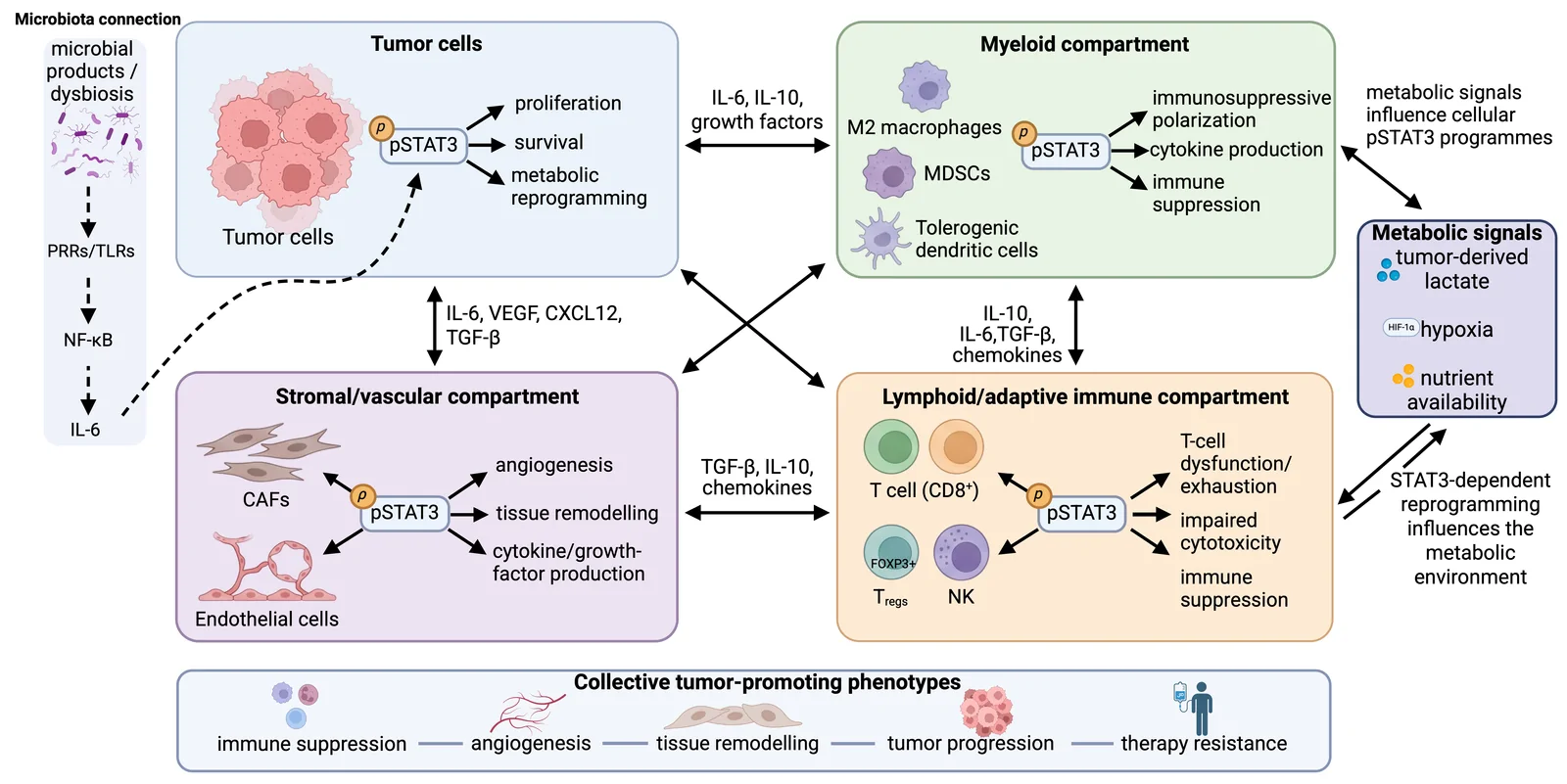
Reciprocal STAT3 signaling across cellular compartments of the tumor microenvironment in cervical, ovarian and endometrial. STAT3 functions as a multicompartmental signaling node linking malignant, myeloid, stromal, vascular, and lymphoid populations within the tumor microenvironment. Persistent STAT3 phosphorylation (pSTAT3) is associated with proliferation, survival, and metabolic reprogramming, while tumor-derived inflammatory and metabolic signals contribute to reciprocal communication with surrounding cells. In the myeloid compartment, STAT3-associated signaling promotes immunosuppressive polarization of M2 macrophages, expansion of myeloid-derived suppressor cells (MDSCs), and tolerogenic dendritic-cell phenotypes, accompanied by production of IL-6, IL-10, growth factors, and other immunoregulatory mediators. In the stromal/vascular compartment, STAT3 signaling in cancer-associated fibroblasts (CAFs) and endothelial cells contributes to angiogenesis, tissue remodeling, and cytokine/growth-factor production, including IL-6, VEGF, CXCL12, and TGF-β. In the lymphoid/adaptive immune compartment, STAT3-associated signaling contributes to T-cell dysfunction and exhaustion, impaired cytotoxicity, Treg accumulation, and reduced NK-cell activity in the context of persistent inflammatory and metabolic stress. Metabolic signals, including tumor-derived lactate, hypoxia, and altered nutrient availability, influence STAT3-associated cellular programs across compartments, while STAT3-dependent cellular reprogramming can further shape the metabolic microenvironment. A potential microbiota connection is also illustrated, whereby microbial products associated with dysbiosis may activate pattern-recognition receptors and Toll-like receptors, leading to NF-κB activation and IL-6 production that can converge on STAT3 signaling; the causal contribution of this axis to carcinogenesis remains to be established. Bidirectional arrows indicate reciprocal communication between cellular compartments. The collective interaction of these pathways contributes to an immunosuppressive and tumor-promoting microenvironment characterized by immune suppression, angiogenesis, tissue remodeling, tumor progression, and therapy resistance. Abbreviations: STAT3, signal transducer and activator of transcription 3; pSTAT3, phosphorylated STAT3; PRRs, pattern-recognition receptors; TLRs, Toll-like receptors; NF-κB, nuclear factor kappa B; IL-6, interleukin-6; IL-10, interleukin-10; VEGF, vascular endothelial growth factor; CXCL12, C-X-C motif chemokine ligand 12; TGF-β, transforming growth factor beta; CAFs, cancer-associated fibroblasts; M2, macrophage phenotype associated with immunosuppressive and tissue-remodeling functions; MDSCs, myeloid-derived suppressor cells; CD8+, cluster of differentiation 8-positive; Tregs, regulatory T cells; FOXP3, forkhead box P3; NK, natural killer; HIF-1α, hypoxia-inducible factor 1-alpha. Created in BioRender. Larco, J.A.L. (2026).

Among stromal populations, CAFs have emerged as major regulators of the inflammatory tumor microenvironment. Activated fibroblasts produce high levels of IL-6, CXCL12, VEGF, TGF-β and matrix-remodeling enzymes, establishing paracrine signaling circuits that promote tumor cell proliferation, epithelial-to-mesenchymal transition (EMT), angiogenesis and therapeutic resistance. IL-6 is particularly relevant because it contributes to sustained JAK/STAT3 activation in both malignant cells and neighboring stromal populations, reinforcing inflammatory feedback loops that become progressively independent of the initiating oncogenic event [61,62] (Figure 3).

The contribution of CAFs appears particularly relevant in ovarian cancer, where progression through the peritoneu results extensive stromal remodeling, including substantial accumulation of carcinoma-associated fibroblasts, largely arising from mesothelial-to-mesenchymal transition, that accompanies disease progression and metastatic dissemination throughout the peritoneal cavity [63]. In addition to producing inflammatory cytokines, CAFs modify extracellular matrix stiffness and organization, influencing both tumor cell invasion and immune cell trafficking. Similar observations have been reported in cervical cancer, where fibroblast activation contributes to progressive remodeling of the cervical stroma during HPV-associated carcinogenesis [64]. Moreover, in endometrial cancer model, a CD146-positive CAF subpopulation similarly sustains STAT3 signaling: CD146^+^ CAF-derived IL-10 activates the JAK1/STAT3 pathway in tumor cells, promoting angiogenesis and vasculogenic mimicry, being associated with poor prognosis [65]. Although the composition of the stromal compartment differs among gynecological malignancies, chronic inflammatory signaling involving STAT3 represents a common feature associated with disease progression.

Endothelial cells also actively participate in this communication network. Beyond their role in angiogenesis, activated endothelial cells regulate leukocyte recruitment, vascular permeability and local cytokine gradients. VEGF-driven angiogenesis, frequently associated with persistent STAT3 activation, generates structurally abnormal vasculature that contributes to hypoxia, heterogeneous drug delivery, high interstitial pressure and impaired immune cell infiltration [66]. These vascular abnormalities further reinforce metabolic stress and inflammatory signaling, illustrating the close relationship between stromal remodeling and immune regulation (Figure 3).

Collectively, these observations indicate that stromal cells should not be regarded merely as supporting elements of the tumor microenvironment. Instead, they actively participate in reciprocal signaling circuits that sustain chronic inflammation and influence the composition and function of immune infiltrates. Within these networks, STAT3 contributes to integrating inflammatory and metabolic signals originating from both malignant and stromal compartments.

### 3.2. Myeloid Cells: A Central Interface Between Metabolism and Immune Suppression

Macrophages, monocytes, dendritic cells, neutrophils and myeloid-derived suppressor cells continuously adapt their phenotype according to cytokine availability, oxygen tension and nutrient composition, making them highly sensitive to changes occurring within the tumor microenvironment. Consequently, these populations occupy a central position that links metabolic adaptation and immune regulation. In the TME, STAT3 promotes M2-like macrophages differentiation, induces the mobilization of myeloid-derived suppressor cells, drives a tolerogenic phenotype in dendritic cells while impairing their maturation, stimulates angiogenesis and epithelial–mesenchymal transition (Figure 3) [24,25]. Moreover, STAT3 transcriptional targets such as arginase 1, PD-L1 (Programmed Death-Ligand 1), IDO (indoleamine-2,3-dioxygenase) and VEGF are part of the mechanisms by which this factor controls the TME [15,39,66,67,68].

Tumor-associated macrophages (TAMs) are prominent components of the tumor microenvironment in cervical, ovarian and endometrial cancers, although their abundance and functional states vary across tumor types and disease contexts. Rather than existing as discrete M1 or M2 subsets, macrophages display remarkable functional plasticity and frequently acquire intermediate activation states shaped by the local microenvironment. Exposure to IL-6, IL-10, TGF-β, CSF-1, lactate and hypoxia collectively promote phenotypes associated with tissue remodeling, angiogenesis and suppression of adaptive immune responses [15].

In cervical cancer, tumor-derived IL-6 and Prostaglandin E2 induce M2 polarization, which correlates with PD-L1 expression and poor prognosis [69]. In ovarian cancer cells, there is evidence that STAT3 is part of a crosstalk mechanism, where tumor cells induce the M2 phenotype, characterized by CD163, IL-6 and IL-10 expression, dependent on STAT3 activity, while macrophages on the other hand promote cell proliferation, through Cyclin D1 expression [18,70]. Furthermore, macrophages frequently constitute one of the dominant immune populations within malignant ascites. These cells produce cytokines and growth factors that support tumor growth while simultaneously limiting effective antitumor immunity. Importantly, these observations reinforce the concept that STAT3 functions within a broader regulatory network rather than acting as an isolated determinant of macrophage behavior.

Myeloid-derived suppressor cells (MDSCs) further contribute to this immunosuppressive landscape. Expansion of MDSCs has been reported in both ovarian and cervical cancers and is associated with advanced disease, impaired T-cell responses and unfavorable clinical outcome [71,72]. Multiple inflammatory mediators contribute to MDSC recruitment and activation, including GM-CSF, G-CSF, IL-6 and VEGF, many of which signal through STAT3 intersecting pathways (Figure 3). Once established within the tumor microenvironment, MDSCs suppress cytotoxic lymphocyte function through mechanisms involving arginine depletion, reactive oxygen species production, nitric oxide synthesis and immunosuppressive cytokine secretion [73]. In patients with endometrial cancer, MDSC expansion has similarly been associated with a systemic inflammatory response, including thrombocytosis, leukocytosis and neutrophilia, and correlates with impaired antitumor immunity [74].

Dendritic cells are similarly influenced by the metabolic and inflammatory characteristics of the tumor microenvironment. Persistent exposure to IL-6, VEGF and lactate impairs dendritic cell maturation, antigen presentation and migration to draining lymph nodes, reducing the induction of effective adaptive immune responses. Experimental studies suggest that STAT3 contributes to maintaining immature or tolerogenic dendritic cell phenotypes, although these effects are strongly influenced by additional environmental factors, including hypoxia and metabolic stress [75]. Consequently, alterations in dendritic cell function should be interpreted as the result of an integrated signaling rather than STAT3 activation alone [75].

Neutrophils have also attracted increasing attention in ovarian cancer, where increased neutrophil infiltration and elevated neutrophil-to-lymphocyte ratios have been associated with poor prognosis [76]. Like other myeloid populations, neutrophils undergo functional reprogramming in response to inflammatory cytokines and metabolic alterations present within the tumor microenvironment. While the precise contribution of STAT3 to neutrophil biology remains incompletely understood, evidence suggests that STAT3 signaling influences neutrophil survival, recruitment and production of inflammatory mediators, further illustrating its participation in multiple components of the myeloid compartment. Moreover, recent evidence indicates that STAT3 is part of the tolerogenic mechanism that neutrophils play a role in in the TME [77].

Taken together, these observations position myeloid cells as a critical interface connecting metabolic adaptation with immune suppression. Rather than responding independently to isolated cytokines or metabolites, these populations integrate multiple environmental signals that collectively shape antitumor immunity. Because STAT3 participates in several of these interconnected pathways, modulation of its activity has the potential to influence not only tumor cells but also the broader immune landscape.

### 3.3. Adaptive Immunity: STAT3 Shapes T-Cell Function Within the TME

The effectiveness of antitumor immunity depends on the ability of adaptive immune cells to recognize and eliminate malignant cells. However, T-cell responses develop within a microenvironment characterized by chronic inflammation, metabolic stress, hypoxia and persistent antigen exposure, conditions that progressively impair effector function while favoring immune tolerance. Rather than resulting from a single immunosuppressive mechanism, adaptive immune dysfunction reflects the cumulative influence of multiple stromal, metabolic and inflammatory signals acting simultaneously throughout tumor progression.

Among these mechanisms, chronic activation of cytokine signaling pathways has emerged as an important determinant of T-cell differentiation. Persistent exposure to IL-6, IL-10 and TGF-β alters the balance between effector and regulatory lymphocyte populations, while continuous antigen stimulation promotes progressive functional exhaustion characterized by reduced proliferative capacity, diminished cytokine production and increased expression of inhibitory receptors (Figure 3) [78]. The relationship between STAT3 and regulatory T cells (Tregs) illustrates this complexity. Multiple studies have associated constitutive STAT3 activation with increased recruitment or maintenance of immunosuppressive lymphocyte populations, although the mechanisms involved differ among tumor types and remain fautily understood [79]. Importantly, Treg accumulation rarely depends on STAT3 signaling alone but instead reflects a coordinated activity of inflammatory cytokines, chemokines and metabolic mediators present within the tumor microenvironment.

Cytotoxic CD8+ T lymphocytes are similarly influenced by the metabolic characteristics of the tumor microenvironment. Nutrient deprivation, hypoxia and extracellular acidification collectively impair T-cell activation and effector function while limiting proliferation and cytokine production. Lactate accumulation has received particular attention because elevated extracellular concentrations reduce T-cell motility, interfere with glycolytic metabolism and diminish cytotoxic activity [54]. Although these effects involve multiple molecular pathways, STAT3-mediated inflammatory signaling contributes to maintaining the immunosuppressive environment that favors T-cell dysfunction (Figure 3).

Natural killer (NK) cells undergo functional alterations during tumor progression in ovarian cancer. In advanced disease, reduced cytotoxicity, impaired cytokine production, and diminished NK cell infiltration have been reported and associated with an immunosuppressive tumor microenvironment [80]. Similar to T lymphocytes, NK-cell activity is influenced by cytokines, metabolites and stromal interactions that collectively shape the local immune landscape. Rather than acting directly on NK cells in isolation, STAT3 contributes to the broader inflammatory milieu that determines NK-cell activation and persistence within tumors [39].

These observations highlight an important principle of tumor immunology. Adaptive immune suppression is not generated by individual signaling pathways acting independently rather emerges from continuous communication among tumor cells, stromal populations and innate immune cells (Figure 3). Within this network, STAT3 participates in several regulatory circuits that collectively influence lymphocyte recruitment, differentiation and function, reinforcing the interconnected nature of tumor–microenvironment communication.

## 4. STAT3 Potential Signalling Convergence Node Between Microbial Dysbiosis and Gynecological Tumor-Promoting Inflammation

The female reproductive tract (FRT) microbiota has emerged as an important modulators of gynecologic health. While the lower FRT (vagina and cervix) of healthy reproductive-age women is characterized by a low-diversity microbial environment dominated by *Lactobacillus* species, the upper FRT (uterus, fallopian tubes and ovaries) harbours a substantially lower microbial biomass and greater microbial diversity [81]. A healthy microbiota supports mucosal barrier function and helps regulate host immunity, whereas dysbiosis may impair barrier integrity and disrupt immune and metabolic homeostasis. These alterations may promote chronic inflammation and cytokine production. Carcinogenesis may therefore arise indirectly from altered host defence responses to dysbiotic microbiota, pathobionts, and/or pathogens, contributing to gynecological cancer development [82]. Importantly, however, the available evidence across gynecological malignancies supports associations between microbial alterations and tumour-relevant inflammatory processes, but does not yet establish a microbiota-driven carcinogenic mechanism.

In cervical cancer, progression from low-grade lesions to high-grade squamous intraepithelial lesions (HSIL) and invasive cervical cancer (ICC) is associated with *Lactobacillus* depletion and increased vaginal microbiota diversity [83]. Specific anaerobic taxa have been identified as potential biomarkers of HSIL and ICC, notably *Sneathia*, *Anaerococcus tetradius*, and *Peptostreptococcus anaerobius.* Conversely, *L. crispatus* dominance has been associated with high-risk HPV clearance and spontaneous regression of dysplastic lesions [83,84]. Although epidemiological studies have consistently linked vaginal dysbiosis with HPV persistence and cervical neoplasia, the mechanisms underlying the bidirectional interaction between HPV and the vaginal microbiota remain incompletely understood. Experimental studies have further demonstrated bidirectional interactions between HPV16 and the vaginal microbiota. HPV16 can alter the vaginal microbial environment by downregulating host mucosal innate peptides that support *Lactobacillus* growth, while organotypic models indicate that HPV16-positive cervical epithelium can enhance the persistence of Sneathia vaginalis following bacterial exposure [85,86]. However, whether these interactions directly promote HPV persistence or drive cervical carcinogenesis remains to be fully established.

In endometrial cancer, alterations in the reproductive-tract microbiota, including the co-occurrence of *Atopobium vaginae* and *Porphyromonas somerae*, particularly in combination with an abnormal vaginal pH (>4.5), have been associated with endometrial hyperplasia and cancer [87,88]. Experimental studies provide further support for a potential functional role of these bacteria: *Porphyromonas somerae* exhibits enhanced growth in the presence of 17β-estradiol and can invade endometrial adenocarcinoma cells in vitro, while *A. vaginae* and *Porphyromonas* spp. have been shown to induce pro-inflammatory responses in endometrial epithelial cells [87,88].

In ovarian cancer, distinct microbial signatures have been identified in tumour tissue, including enrichment of microorganisms such as *Brucella*, *Mycoplasma*, and *Chlamydi* [89]. In parallel, a multicentre case–control study identified an association between ovarian cancer and a cervicovaginal microbiota characterized by reduced *Lactobacillus* abundance, indicating that ovarian cancer may be accompanied by alterations in the local microbial community, particularly among women younger than 50 years Together, these findings indicate an association between microbial alterations at different reproductive-tract sites and ovarian cancer [90].

A potential mechanistic link to STAT3 may involve inflammatory signalling induced by microbial products. Recognition of microbial components through pattern-recognition receptors, including Toll-like receptors, can activate NF-κB and promote the production of cytokines such as IL-6, an established upstream activator of JAK/STAT3 signalling (Figure 3). Consistent with this pathway, IL-6/STAT3 signalling has been shown to promote tumor-associated phenotypes in cervical, endometrial and ovarian cancers. In cervical cancer, HPV-driven NF-κB activation induces IL-6 production and subsequent STAT3 activation, supporting tumour-cell proliferation and survival [91]. In endometrial cancer, IL-6-dependent STAT3 activation promotes tumour-cell proliferation, migration and invasion, whereas inhibition of this pathway attenuates these effects [35,38]. Importantly, experimental evidence provides a direct link between a microbial product and this pathway in ovarian cancer: LPS-treated cancer-associated fibroblasts activate NF-κB and secrete IL-6, which promotes ovarian cancer-cell malignancy through JAK2/STAT3 signalling [92].

Together, these findings support a model in which dysbiosis or exposure to microbial products may generate inflammatory signals that converge on IL-6/JAK/STAT3 signalling and thereby influence tumour-promoting cellular responses. However, the causal contribution of this microbiota–IL-6–STAT3 axis to gynaecological carcinogenesis remains to be established in vivo (Figure 3).

## 5. Clinical Translation of STAT3-Targeted Therapies

The strong biological rationale supporting STAT3 as a therapeutic target has driven extensive drug development efforts over the past two decades [93,94]. Nevertheless, translating this rationale into effective clinical therapies has proven challenging, reflecting the context-dependent functions of STAT3 across tumor, immune, and stromal compartments, as well as difficulties in achieving sufficient target engagement and therapeutic selectivity. These challenges have led to the development of diverse approaches to modulating STAT3 signaling, ranging from indirect inhibition of upstream regulators, such as JAK1/2, to direct STAT3 inhibitors, antisense oligonucleotides, and targeted protein degraders. Because these strategies differ substantially in their molecular specificity and biological consequences, distinguishing direct STAT3 targeting from indirect pathway modulation is essential when interpreting their preclinical and clinical evidence.

### 5.1. Therapeutic Strategies Targeting the STAT3 Pathway

Several complementary approaches have been developed to modulate STAT3 signaling, ranging from indirect pathway inhibition to direct STAT3 blockade and targeted protein degradation. Early strategies focused on indirect inhibition of STAT3 through blockade of upstream kinases, particularly JAK family members. Although JAK inhibition can reduce STAT3 phosphorylation, JAK proteins regulate multiple cytokine and growth factor pathways, and their inhibition therefore affects signaling networks beyond STAT3. Consequently, responses to JAK inhibitors cannot be attributed specifically to STAT3 blockade [8]. Subsequent strategies sought to inhibit STAT3 more directly by preventing SH2-domain-mediated dimerization, thereby blocking nuclear translocation and transcriptional activity. Although several small molecules demonstrated encouraging preclinical activity, limitations related to specificity, pharmacokinetics and intracellular target engagement slowed clinical translation [89,91]. Antisense oligonucleotides provide an alternative strategy by reducing STAT3 expression rather than directly inhibiting its activity. More recently, targeted protein degraders have introduced a distinct therapeutic concept by promoting STAT3 ubiquitination and proteasomal degradation. Together, these strategies illustrate the evolution of STAT3-targeted therapy from indirect pathway modulation toward increasingly direct and sustained target suppression.

### 5.2. Clinical Development of STAT3-Targeted Therapies

Clinical development of STAT3-targeted therapies has provided important evidence of pharmacological target engagement and biological activity, but clinical efficacy has generally been modest and heterogeneous across tumor types and treatment settings (Table 1).

Early clinical experience with direct STAT3 inhibitors exposed several pharmacological barriers. OPB-31121, an oral STAT3 inhibitor evaluated in a phase I study of patients with advanced solid tumors, demonstrated limited antitumor activity and an unfavorable pharmacokinetic profile. No objective responses were observed, and drug exposure varied substantially among patients, with pharmacokinetic findings suggesting insufficient exposure relative to concentrations associated with antitumor activity in preclinical models [95]. OPB-51602 subsequently provided evidence that pharmacological inhibition of STAT3 could achieve target modulation in patients with refractory solid tumors, with inhibition of STAT3 Y705 phosphorylation demonstrated in peripheral blood mononuclear cells. However, its clinical development was constrained by gastrointestinal toxicities and peripheral neuropathy, and antitumor activity was limited [96]. In a separate phase I study in patients with relapsed/refractory hematological malignancies, OPB-51602 was associated with peripheral sensory neuropathy, gastrointestinal toxicities, and metabolic adverse events, including lactic acidosis; no objective responses were observed [97].

Danvatirsen (AZD9150), a STAT3-targeting antisense oligonucleotide, has been evaluated in multiple clinical settings. Antisense-mediated STAT3 suppression provided a different approach by reducing STAT3 expression rather than directly inhibiting its activity. In a phase II trial evaluating danvatirsen combined with durvalumab (anti-PD-L1) in advanced pancreatic ductal adenocarcinoma (PDAC), non-small-cell lung cancer (NSCLC), and mismatch repair-deficient colorectal cancer (MRD-CRC), no objective responses were observed [98]. Parallel preclinical analyses further showed that danvatirsen-mediated STAT3 suppression affected pancreatic stellate cells and myeloid populations without uniformly reversing the immunosuppressive tumor microenvironment, highlighting the complexity of targeting STAT3 within the tumor microenvironment [98]. Likewise, danvatirsen combined with acalabrutinib, an irreversible covalent inhibitor of Bruton tyrosine kinase (BTK), was evaluated in patients with relapsed/refractory diffuse large B-cell lymphoma and results in a modest objective response rate with a short median response duration [99].

More recent clinical development of direct STAT3 inhibition has provided stronger evidence of pharmacological target engagement. TTI-101 (formerly C-188-9) is an orally bioavailable small-molecule inhibitor that binds the STAT3 SH2 domain and inhibits STAT3-mediated transcription. In the first-in-human phase I study (NCT03195699), 64 patients with advanced solid tumors were treated. Among 41 patients evaluated for response, five (12%) achieved confirmed partial responses and 17 (41%) had stable disease. Treatment was generally well tolerated, with diarrhea being the most frequent treatment-related adverse event and no dose-limiting toxicities or treatment-related deaths were reported. Importantly, paired tumor biopsies demonstrated reductions in phosphorylated STAT3 in patients with detectable pSTAT3 at baseline, providing pharmacodynamic evidence of target engagement [100].

Moreover, targeted protein degradation represents a further evolution of STAT3-directed therapy. KT-333 is a STAT3-directed protein degrader designed to promote STAT3 degradation rather than transiently inhibit its signaling activity. The first-in-human phase 1a/1b study (NCT05225584) evaluated KT-333 in patients with relapsed or refractory hematologic malignancies and solid tumors, incorporating pharmacokinetic and pharmacodynamic assessments of STAT3 protein depletion. Preliminary clinical data demonstrated robust, dose-dependent STAT3 degradation in peripheral blood mononuclear cells, while clinical efficacy remained limited at the reported data cutoff [101]. This approach is particularly relevant to the clinical translation of STAT3 targeting because it tests whether sustained depletion of the target can be achieved in patients rather than simply inhibiting a specific signaling event.

Collectively, these studies illustrate the evolution of STAT3-targeted drug development from early small-molecule inhibitors toward approaches designed to improve target specificity, pharmacodynamic activity, and durability of pathway suppression. However, the limited and heterogeneous clinical responses observed to date indicate that target engagement alone may be insufficient to predict therapeutic benefit, emphasizing the importance of disease context, biomarker selection, and rational combination strategies.

### 5.3. Biomarker Challenges and Lessons from Clinical Studies

Despite compelling preclinical evidence supporting STAT3 as a therapeutic target, clinical translation has remained disappointing. After nearly three decades of drug development efforts, no STAT3 inhibitor has received FDA approval for cancer treatment, and only a limited number of compounds have advanced to clinical testin. These findings reveal a substantial disconnect between biological rationale and clinical efficacy.

A central obstacle to the clinical translation of STAT3-targeted therapies lies in the absence of a validated, standardized biomarker for patient stratification. Three candidate markers have been proposed, total STAT3, phosphorylated STAT3 (pSTAT3), and circulating IL-6, yet each reflects a distinct and context-dependent dimension of pathway activity. Beyond prognostic inconsistency, pSTAT3 measured by IHC is plagued by substantial technical variability: differences in antibody clones, fixation protocols, staining platforms, and the absence of a universally validated positivity cutoff generate discordant results across laboratories, making multicenter biomarker harmonization extremely difficult [102]. This instability extends to the tumor itself: Marginean and collaborators evaluated pSTAT3 expression by immunohistochemistry in paired primary colorectal tumors and corresponding liver metastases from 103 patients with metastatic disease. Although pSTAT3 expression was frequently detected in both primary tumors (71%) and metastatic lesions (76%), substantial discordance was observed between paired samples. Differences in pSTAT3 staining patterns were identified in 64% of cases, including loss of expression in 28% and gain of expression in 36% of liver metastases relative to the primary tumor. Furthermore, agreement between primary and metastatic sites was extremely poor, indicating that pSTAT3 activation status may change dynamically during tumor progression and metastatic dissemination [103]. In the same way, a meta-analysis of 4,513 breast cancer patients found no consistent relationship between STAT3 or pSTAT3 expression and overall survival, with paradoxical subgroup findings in which pSTAT3 overexpression associated with improved breast cancer-specific survival, underscoring that these markers carry divergent and tumor-context-dependent prognostic signals [104].

Circulating IL-6, meanwhile, behaves as a dynamic signal rather than a static stratification tool: IL-6 levels rose significantly with disease progression in patients treated with CDK4/6 inhibitors, and STAT3 inhibition was effective only in models where tumor-cell pSTAT3 was already detectable at baseline, highlighting that IL-6 reflects a temporally variable state rather than a fixed biomarker of pathway dependency [105]. Compounding these issues, no validated transcriptomic signature for “STAT3-activated tumors” exists across cancer types, a gap that is even more pronounced in gynecological cancers, where large-scale molecular profiling efforts defining STAT3-dependent subtypes remain absent, leaving clinicians without a reliable tool to identify patients most likely to benefit from pathway-targeted intervention.

Moving forward, it is increasingly clear that isolated pSTAT3 measurements are insufficient to capture the complexity of STAT3 biology in the TME. A composite biomarker panel, integrating tumor-cell pSTAT3, immune-compartment pSTAT3, serum IL-6, and PD-L1 expression, may offer a more biologically faithful strategy for patient stratification. Critically, future trials must address not only which analyte to measure, but in which compartment, by which validated platform, and at which threshold, since these choices have proven to be as consequential as the therapeutic intervention itself.

Another important lesson concerns patient heterogeneity. Constitutive STAT3 activation is not uniformly present across all tumors, nor does it necessarily reflect the same biological processes in different patients. Variability in cytokine production, stromal composition, immune infiltration and metabolic status may substantially influence dependence on STAT3 signaling. Consequently, therapeutic responses are likely to depend on biological context rather than pathway activation alone.

Finally, accumulating clinical experience reinforces an increasingly important concept in oncology: signaling pathways rarely function independently. Tumor progression is sustained by dynamic regulatory networks capable of compensating for inhibition of individual components. Consequently, therapeutic modulation of STAT3 is unlikely to eliminate all mechanisms supporting tumor growth but may substantially influence the broader signaling landscape when combined with complementary therapeutic strategies.

### 5.4. Clinical Evidence in Gynecological Cancers

Clinical evidence for STAT3-targeted therapies in gynecological cancers remains considerably more limited than that available for other solid tumors. Moreover, the available studies differ substantially in their degree of STAT3 specificity, ranging from direct STAT3 inhibition to indirect modulation of upstream cytokine or kinase pathways. Among gynecological malignancies, ovarian cancer has received the greatest clinical attention, whereas clinical evidence in cervical and endometrial cancers remains scarce (Table 2).

In ovarian cancer cases, direct clinical evaluation of STAT3 targeting has been limited. Danvatirsen (AZD9150) was evaluated in the phase II NCT02417753 study in patients with metastatic ovarian or gastrointestinal malignancies associated with malignant ascites. However, the trial was terminated because of poor accrual after enrollment of only one patient, precluding meaningful assessment of clinical efficacy. Thus, despite the biological rationale for STAT3 targeting in ovarian cancer, this study does not provide evidence for clinical efficacy. Similarly, JAK inhibition has been investigated as an indirect approach to modulate STAT3 signaling in ovarian cancer patients. Ruxolitinib, a JAK1/2 inhibitor, was evaluated in combination with carboplatin and paclitaxel in patients with advanced epithelial ovarian, fallopian tube, or primary peritoneal cancer (NCT02713386). Because JAK1/2 regulate multiple cytokine-dependent pathways, however, the biological effects of ruxolitinib cannot be attributed specifically to STAT3 inhibition. In ovarian cancer, clinical studies have also explored indirect or emerging approaches to modulate STAT3 signaling. Ruxolitinib, a JAK1/2 inhibitor, was evaluated in the NRG-GY007 phase I/II trial in patients with advanced epithelial ovarian, fallopian tube, or primary peritoneal cancer; however, its effects cannot be attributed specifically to STAT3 because JAK1/2 regulate multiple cytokine-dependent pathways [106]. More recently, the repurposed drug atovaquone has entered a phase II trial (NCT05998135) in platinum-resistant ovarian cancer based on preclinical evidence suggesting STAT3 inhibition [107]. In cervical cancer, clinical evidence for STAT3-directed therapy remains lacking. Although persistent STAT3 activation has been associated with tumor progression, immune evasion, and therapeutic resistance in preclinical and translational studies, direct clinical trials evaluating STAT3 inhibitors in cervical cancer have not yet established therapeutic efficacy. This gap is particularly relevant given the strong influence of HPV-associated inflammation and immune remodeling on the cervical tumor microenvironment. Similarly, clinical evidence for STAT3-targeted therapy in endometrial cancer remains limited. Available studies have predominantly investigated STAT3 activation as a biological or prognostic feature, whereas therapeutic studies have largely remained at the preclinical stage.

### 5.5. STAT3-Targeted Combination Strategies: Immunotherapy and Metabolic Vulnerabilities

The pleiotropic nature of STAT3 opens immunotherapy treatment opportunities. Due to its role in tumor cells and immune cells in the TME, STAT3 targeting can be used for immune response modulation and tumor progression control [25].

In advanced or recurrent endometrial cancer, phase III trials such as NRG-GY018 and RUBY have established the clinical benefit of incorporating immune checkpoint blockade (ICB) into first-line chemotherapy. In NRG-GY018, pembrolizumab combined with carboplatin–paclitaxel significantly prolonged progression-free survival (PFS) compared with chemotherapy alone in both mismatch repair-deficient (dMMR) and mismatch repair-proficient (pMMR) tumors, with a particularly pronounced benefit in dMMR tumors [108]. Similarly, the RUBY trial demonstrated that dostarlimab plus carboplatin–paclitaxel significantly improved PFS, with the greatest benefit observed in dMMR/microsatellite instability-high (MSI-H) tumors (24-month PFS, 61.4% vs. 15.7%; hazard ratio [HR], 0.28), and an overall survival (OS) benefit in the overall population (24-month OS 71.3% vs. 56.0%; HR, 0.64) [109]. These findings establish ICB as an important component of treatment for advanced or recurrent endometrial cancer while highlighting the molecular heterogeneity of treatment response. In preclinical tumor models, STAT3 silencing combined with anti-PD-L1 treatment resulted in greater tumor regression than STAT3 silencing alone and promoted conversion of an immunosuppressive TME toward a T-cell-enriched phenotype [110]. Similarly, JAK inhibition increased CD8+ T-cell infiltration and restored sensitivity to anti-PD-1 therapy in pancreatic tumor models resistant to ICB [111]. Furthermore, transcriptomic analyses of CD19 CAR-T cells from patients with chronic lymphocytic leukemia demonstrated that enhanced IL-6/STAT3 activity in CAR-T cells correlates with greater expansion, persistence, and antitumor activity, indicating that STAT3 signaling is necessary for the efficacy of CAR-T cell-based therapy [112]. Still, the correlation between STAT3 signaling and immune checkpoint molecules provides a promising strategy to improve the efficacy of current immunotherapies.

The context dependence of STAT3 signaling becomes particularly apparent across different gynecological malignancies. In cervical cancer models, our group demonstrated in an HPV-associated tumor model that pharmacological inhibition of STAT3 impaired tumor growth while enhancing tumor-specific T-lymphocyte responses [113]. Similarly, ovarian tumors display multiple immune evasion mechanisms including recruitment of regulatory T lymphocytes, M2 macrophages, and PD-L1 expression. Based on this, IL-6 blockade with siltuximab showed modest clinical activity but achieved prolonged disease stabilization in recurrent, platinum-resistant cases, with better responses in patients with higher IL-6 expression [114].

PARP inhibitors (PARPi), such as olaparib, are particularly effective in BRCA1/2-deficient ovarian cancer, but acquired resistance remains a major clinical challenge. In ovarian cancer models and matched patient samples, PARPi treatment was shown to increase STAT3 activation not only in tumor cells but also in tumor-associated immune cells and fibroblasts [115]. In BRCA1-deficient ovarian cancer models, olaparib-induced STAT3 activation was associated with increased IL-6/JAK/STAT3 signaling and production of chemokines including CCL2, CCL20, CX3CL1, and CXCL1, which promoted the accumulation and polarization of immunosuppressive macrophages. Genetic depletion of STAT3 reduced macrophage polarization, increased tumor-infiltrating T cells, and restored sensitivity to olaparib in vivo [11].

These findings provide a rationale for targeting the immunosuppressive microenvironment in addition to tumor-intrinsic mechanisms of PARPi resistance. STING activation represents a particularly promising complementary strategy. In BRCA1-deficient ovarian cancer models, olaparib-induced STING-dependent innate and adaptive immune responses, which were further enhanced by PD-1 blockade [116]. More recently, STING agonism was shown to remodel the immunosuppressive microenvironment in PARPi-resistant ovarian cancer, restoring sensitivity to PARP inhibition, with further increased antitumor activity when combined with PD-1 blockade [11]. Together, these findings support the investigation of combined strategies targeting PARPi-induced adaptive resistance, myeloid immunosuppression, and immune checkpoint signaling, although their clinical efficacy remains to be established.

The close relationship between metabolism and immune regulation makes metabolic pathways attractive therapeutic partners for STAT3-targeted interventions. Tumor-associated lactate accumulation, hypoxia, glutamine metabolism, and lipid remodeling influence both malignant cells and immune populations, creating a metabolically restrictive environment that limits effective antitumor immunity. Among these approaches, inhibition of lactate transport through monocarboxylate transporters, modulation of glutamine utilization, and interventions targeting tumor hypoxia have attracted increasing attention. However, relatively few studies have directly evaluated these strategies in combination with STAT3 inhibition, and their potential in cervical, ovarian or endometrial cancers remains to be established.

## 6. Future Perspectives

Despite substantial progress in understanding STAT3 signaling, several questions remain unresolved. Future studies should better define the spatial and temporal dynamics of STAT3 activation and determine how these patterns vary according to tumor type, molecular subtype, and cellular compartment. In particular, integrating spatial transcriptomics, multiplex imaging, and single-cell approaches may help resolve the heterogeneity of STAT3 activity within individual tumors and identify cellular populations that are most dependent on this pathway.

An important priority will be the identification of robust biomarkers capable of predicting sensitivity to STAT3-targeted therapies and monitoring pharmacodynamic responses. Combining molecular, metabolic, and immune profiling may help identify patients who are most likely to benefit from STAT3 inhibition or from specific combination strategies. Further investigation is also needed to determine whether metabolic or immune interventions can selectively enhance STAT3-targeted therapies while limiting toxicity to normal tissues.

Rather than pursuing a universal therapeutic approach, future research should therefore focus on defining tumor- and context-specific vulnerabilities associated with STAT3 signaling. This may ultimately enable more precise therapeutic strategies for cervical, ovarian, and endometrial cancers.

## 7. Conclusions

Overall, the evidence reviewed here supports STAT3 as an important signaling node linking tumor-intrinsic processes with metabolic, immune, and stromal components in cervical, ovarian, and endometrial cancers. Across these tumor types, STAT3 has been implicated in tumor progression, immune regulation, metabolic adaptation, and therapeutic resistance. However, these functions are highly context dependent and may vary according to tumor type, molecular characteristics, and cellular compartment. Thus, STAT3 should not be interpreted as a uniform driver across these cancers, but rather as a context-dependent component of interconnected signaling networks within the tumor microenvironment.

Taken together, the evidence supports continued investigation of STAT3 as a therapeutic target while emphasizing the need to consider the specific biological context of each tumor. Moving beyond the concept of STAT3 as an isolated oncogenic pathway may help refine therapeutic approaches and identify settings in which STAT3-directed strategies are most likely to provide meaningful benefit. Ultimately, a more precise understanding of STAT3 function across cervical, ovarian, and endometrial cancers will be essential for translating mechanistic insights into clinically relevant therapeutic strategies.

## Figures and Tables

**Table 1:** Selected clinical development of STAT3-targeted therapies across solid and hematologic malignancies. The table summarizes representative clinical studies evaluating direct STAT3-targeted approaches, including small-molecule inhibitors, antisense oligonucleotides (ASOs), and targeted protein degraders, as well as selected combination strategies. Direct STAT3 targeting refers to approaches designed to inhibit or deplete STAT3 itself, whereas combination strategies may additionally modulate other signaling pathways. Abbreviations: ASO, antisense oligonucleotide; BTKi, Bruton tyrosine kinase inhibitor; cfDNA, circulating cell-free DNA; DLBCL, diffuse large B-cell lymphoma; DOR, duration of response; EGFR, epidermal growth factor receptor; GI, gastrointestinal; ICB, immune checkpoint blockade; MRD-CRC, mismatch repair-deficient colorectal cancer; NSCLC, non-small-cell lung cancer; ORR, objective response rate; PD, pharmacodynamic; PDAC, pancreatic ductal adenocarcinoma; PR, partial response; R/R, relapsed/refractory; SD, stable disease; STAT3, Signal Transducer and Activator of Transcription.

Agent	Modality	STAT3 Targeting	Trial	Population	Main Findings	PD Biomarker	Main Limitation
OPB-31121	Small molecule	Direct	Phase I	Advanced solid tumors	No objective responses in one study; limited SD in another	Limited/ variable	GI toxicity, inadequate exposure
OPB-51602	Small molecule	Direct	Phase I	Solid tumors	2 PRs, both EGFR-mutant NSCLC	↓ pSTAT3-Y705	GI toxicity, neuropathy, pneumonitis
OPB-51602	Small molecule	Direct	Hematologic phase I	R/R hematologic malignancies	No clear response	pSTAT3	Lactic acidosis/neuropathy
Danvatirsen	ASO	Direct	NCT02417753 phase II	Ovarian/GI cancer with malignant ascites	1 patient; terminated	STAT3-related analyses	Poor accrual
Danvatirsen + durvalumab	ASO + ICB	Direct + immune	NCT02983578 phase II	PDAC/NSCLC/MRD-CRC	No objective responses	Tumor biomarkers	Limited efficacy
Danvatirsen + acalabrutinib	ASO + BTKi	Direct + indirect	Phase I	R/R DLBCL	ORR 24%; median DOR 1.9 mo	cfDNA	Limited durability
TTI-101	Small molecule	Direct	NCT03195699 phase I	Advanced solid tumors	5/41 PR; 17/41 SD	↓ pSTAT3	Heterogeneous population
KT-333	Protein degrader	Direct	NCT05225584 phase Ia/Ib	Hematologic + solid tumors	Clinical development of degrader	STAT3 degradation	Early stage development

**Table 2:** Clinical studies evaluating STAT3-targeted or STAT3-associated therapies in gynecological cancers. Selected clinical studies evaluating direct STAT3 inhibition or indirect modulation of the STAT3 signaling axis in ovarian malignancies are summarized. Direct approaches include agents designed to inhibit STAT3 activity or reduce STAT3 expression, whereas indirect approaches target upstream regulators such as IL-6 or JAK1/2 and therefore affect signaling pathways beyond STAT3. Abbreviations: CRP, C-reactive protein; GI, gastrointestinal; IL-6, interleukin-6; JAK, Janus kinase; PD, pharmacodynamic; PFS, progression-free survival; PR, partial response; SD, stable disease; STAT3, Signal Transducer and Activator of Transcription 3; VEGF, vascular endothelial growth factor.

Agent	Modality	STAT3 Target	Phase	Gynecological Cancer Population	Main Findings	PD/Biomarker	Main Limitation/Status
Danvatirsen (AZD9150)	Antisense oligonucleotide	Direct	II	Metastatic ovarian cancer with malignant ascites; GI malignancies also included	Trial terminated after enrollment of only 1 patient; efficacy could not be assessed	STAT3-related immune markers in ascites	Poor accrual; terminated
Siltuximab (CNTO328)	Monoclonal antibody	Indirect—IL-6 blockade	II	Recurrent/platinum-resistant epithelial ovarian cancer	Among 18 evaluable patients, 1 PR by RECIST/CA-125 criteria; 8 SD, including 4 lasting ≥ 6 months; median PFS 12 weeks	IL-6 expression; circulating IL-6-regulated cytokines, CCL2, CXCL12, VEGF	Limited objective activity; single-arm study
Ruxolitinib	JAK1/2 inhibitor	Indirect—JAK/STAT3 modulation	I/II	Stage III–IV epithelial ovarian, fallopian tube, or primary peritoneal cancer	Randomized phase II study; designed to evaluate PFS and OS benefit from adding ruxolitinib to frontline chemotherapy	Exploratory: IL-6, CRP, CD8:FOXP3, CD3/CD4, HLA-I/II, CD68, ALDH/CD133	Completed; effects cannot be attributed specifically to STAT3 because JAK1/2 regulate multiple pathways
Atovaquone	Small molecule	Proposed direct STAT3 inhibition	II	Platinum-resistant ovarian cancer	Clinical trial initiated; early report described 2/28 planned patients enrolled at time of reporting	Pre-/on-treatment STAT3-dependent gene expression; immune profiling	Early clinical development; efficacy not yet established

## Data Availability

No new data were created or analyzed in this study. Data sharing is not applicable to this article.
